# Editorial: Neuroimaging Findings in Sleep Disorders and Circadian Disruption

**DOI:** 10.3389/fneur.2019.00249

**Published:** 2019-03-21

**Authors:** Xi-Jian Dai, Hengyi Rao, Kai Spiegelhalder

**Affiliations:** ^1^Department of Medical Imaging, Jinling Hospital, Medical School of Nanjing University, Nanjing, China; ^2^Division of Sleep, Perelman School of Medicine, University of Pennsylvania, Philadelphia, PA, United States; ^3^Department of Psychiatry and Psychotherapy, Faculty of Medicine, Medical Center – University of Freiburg, Freiburg, Germany

**Keywords:** insomnia, obstructive sleep apnea, sleep deprivation, functional magnetic resonance imaging, sleep disorders, circadian rhythm

Each of us spends almost a third of our life asleep. Thus, obviously, sleep is a necessary physical need in human life. After sleep, the tired nerve cells and the biological characteristics of long-distance signal transmission recover to normal physiological function. In general, precise control of the sleep process is the basis of normal life processes including blood, metabolism, immune, endocrine, and brain activity, and is key to plasticity formation, information processing, and function implementation [(1–5); Dai et al.].

Sleep has played a minor role as object of research for a long time. Yet, recently there is a growing public interest in sleep. Sleep disorders are a major public health problem and widespread in today's society. In modern society, more and more people undergo an increased curtailment of daily sleep because of work overtime, exam preparation, shift working and long-term working or driving, resulting in an increased incidence of sleep disorders. The disturbed and/or interrupted sleep may be associated with a number of clinical conditions and has a detrimental effect on attention, working memory, executive functioning, emotion, or even metabolism. Nowadays, important challenges are posed to sleep disorders for which approved treatments are of limited efficacy.

Although there is surprising upsurge in neuroimaging findings in addressing the brain structural and functional changes associated with sleep disorders and circadian disruption, it is still difficult to glean a consistent story about its neuropathology of brain alterations. Therefore, a more comprehensive understanding of brain structural and functional changes associated with sleep disorders and circadian disruption are needed. The aim of this Research Topic is to contribute to a better understanding of the link between brain and sleep disorders, and offer an up-to-date view on how sleep affects our brain.

## Primary Insomnia

This specific issue includes two studies focusing on insomnia. In one study by Li et al. the authors found decreased effective connectivity from right ventral and dorsal anterior insula to the precuneus, postcentral gyrus, and cerebellum posterior lobe, which negatively correlated with Pittsburgh Sleep Quality Index and Insomnia Severity Index scores.

In another study by Dai et al. the authors found that acute sleep deprivation (SD) and chronic insomnia showed widespread changes in gray matter volumes (GMVs) with shared but also distinct neurobiological representation in brain morphology. Acute SD may be associated with inhibition in sensory-informational processing with decreased GMVs in the somatosensory areas to compensate for the effects of sleep loss on advanced cognitive function, while primary insomnia may be associated with increased GMVs in several brain areas, which may be key a core predisposing or perpetuating factor of ultimately hampering the ability to initiate or maintain sleep.

## SD

This specific issue also includes four studies focusing on SD and one study on narcolepsy. In one study by Satterfield et al. the authors reported that increased baseline responsiveness within reward regions are more vulnerable to SD-induced overeating. Functional activation within the ventral striatum during the multi-source interference task (MSIT) and n-back task positively correlated with total caloric and carbohydrate intake during the final 6 h (06:00–12:00) of acute SD. Activation within the middle and superior temporal gyri during the MSIT also correlated with total carbohydrates consumed.

In the second study by Dai et al. these authors found prolonged acute SD hours (20, 24, 32, 36 h SD) exhibit accumulative brain atrophic effects and recovering plasticity (after one night sleep recovery) on brain morphology, in line with the behavioral changes on attentional and working memory tasks, which may provide the neurobiological basis for attention and memory impairments following sleep loss.

The last two studies focus on finding potential indicators. Chen et al. and Kong et al. found that the amplitude of low-frequency fluctuation and short-range and long-range functional connectivity density may be potential biomarkers to describe the altered regional brain cortical activities and intrinsic brain functional organization disturbed by acute SD with high discriminating performances.

## Obstructive Sleep Apnea

One study by Chen et al. examined topological changes in obstructive sleep apnea and found decreased functional connectivity within the default mode network, which may contribute to the observed topological reorganization of clustering coefficient, path length, global efficiency, and Montreal cognitive assessment score. These findings may provide evidence of cognitive deficits in obstructive sleep apnea.

## Narcolepsy With Cataplexy

One study by Fulong et al. found that both adult and juvenile narcolepsy had lower fractional low-frequency fluctuations (fALFF) values in bilateral medial superior frontal gyrus, bilateral inferior parietal lobule and supra-marginal gyrus, and higher fALFF values in bilateral sensorimotor cortex and middle temporal gyrus. The right medial superior frontal gyrus discriminated between narcolepsy and healthy controls with high degree of sensitivity (100%) and specificity (88.9%), which may suggest that the fALFF may be a helpful imaging biomarker.

## Restless Legs Syndrome

One study by Hermesdorf et al. evaluated the relationship between the genetic risks and subcortical volumes for restless legs syndrome, but neither of them gave rise to the GMV changes in the hippocampal and subcortical shapes.

## Circadian Disruption

The ninth study by McGlashan et al. investigated whether BOLD-fMRI activation of human suprachiasmatic area in response to light in a 30 s block-paradigm of lights on (100 lux) and lights off (<1 lux) is related to a functional outcome. They found a positive correlation between this activation and melatonin suppression, which may help to better understand the clinical vulnerability influenced by circadian disruption.

## Conclusions

Together, this issue features articles that address the relationships between sleep-related disorders and the brain structure and function using neuroimaging methods. We hope this special issue will contribute to a better understanding of the link between brain and sleep disorders and offer an up-to-date view on how sleep affects our brain. We believe that this special issue will stimulate discussions in a wider public involving not only those working in the field, since both conditions cause an extreme impairment of quality of life, in particular in those patients suffering from both conditions.

## Author Contributions

All authors listed have made a substantial, direct and intellectual contribution to the work, and approved it for publication.

### Conflict of Interest Statement

The authors declare that the research was conducted in the absence of any commercial or financial relationships that could be construed as a potential conflict of interest.
